# Effects of Animal Fat Replacement by Emulsified Melon and Pumpkin Seed Oils in Deer Burgers

**DOI:** 10.3390/foods12061279

**Published:** 2023-03-17

**Authors:** Elena Martínez, José E. Pardo, Adrián Rabadán, Manuel Álvarez-Ortí

**Affiliations:** Escuela Técnica Superior de Ingeniería Agronómica y de Montes y Biotecnología, Campus Universitario s/n, 02071 Albacete, Spain

**Keywords:** functional foods, physicochemical analysis, stable oil emulsion, unsaturated fatty acids

## Abstract

Meat products such as burgers contain large amounts of saturated fat and are considered unhealthy foods by a society that is increasingly aware of the impact of food on their health, as there is a widespread idea that the consumption of large amounts of saturated fats is related to cardiovascular diseases, some types of cancer and obesity. The main goal of this study was to reformulate deer burgers by replacing the saturated fat from its composition with emulsions of oil extracted from melon and pumpkin seeds. Three emulsions were made with these oils (guar gum and inulin, sodium alginate and maltodextrin) to obtain a solid texture. Then, burgers were elaborated, using the vegetable oil emulsions to replace partially (50%) or totally (100%) the animal fat usually used in their elaboration. Physical parameters such as color and texture, consumer evaluation, proximate analysis and the fatty acid composition obtained by gas chromatography were analyzed. The burgers made with emulsified oils showed a higher weight loss, but with a minor loss of caliber and hardness (*p* < 0.05). From the sensory point of view, the reformulated burgers were positively valued by consumer judges when external aspect, odor, flavor and texture were evaluated. Furthermore, the addition of oil emulsions results in a lower fat content and in an increment of the proportion of unsaturated fatty acids, especially linoleic acid (*p* < 0.05). The inclusion of emulsified melon and pumpkin oil in deer burgers leads to an increase in the content of polyunsaturated fatty acids in burgers that, although they showed small differences in texture attributes (especially hardness and cohesiveness), were well valued by consumer judges in all sensory attributes evaluated.

## 1. Introduction

World consumption of meat has doubled in the last 20 years due to income and population growth [1], reaching 320 million tons in 2018 [2]. Growth in global consumption of meat over the next decade is projected to increase by 14% by 2030 compared to data from the 2018–2020 period. Within these data, game meat such as deer meat plays a residual role in developed countries but has a complete nutritional profile; additionally, its environmental impact is lower than farmed meat, so it is growing in popularity among consumers [3,4]. Meat products are generally consumed as a source of proteins with high biological value, minerals such as iron or selenium, and vitamins A, B12 and folic acid [5]. In this sense, deer meat presents a high nutritional value; specifically, it has a high content of total protein and water-soluble nitrogen, as well as a low-fat content compared to other types of meat. In addition, deer meat presents good sensory attributes as taste, aroma or tenderness, so it can be perfectly used for different culinary purposes [6]. Furthermore, the consumption of deer meat has become a multisectoral driver of the rural economy [7].

However, some meat products such as burgers are considered unhealthy due to their high fat content, especially saturated fatty acids and cholesterol, which are associated with cardiovascular diseases, some types of cancer and obesity [8], although recent works have questioned these adverse effects of animal fats [9,10]. Nevertheless, the presence of fat in these meat products is essential, since it plays a crucial role due to its relationship with several sensory attributes, such as flavor or juiciness, very appreciated by consumers. In addition, fat contributes to other processing and technological characteristics of the burgers, as it is related to the rheological properties of the mixture, the cooking loss and the water holding capacity, which make burgers more palatable [11]. Therefore, the current food industry faces the challenge of developing new ways to introduce healthy fats into processed meat products while maintaining their physical and sensory characteristics.

The substitution of solid fats for unsaturated oils usually affects the texture of the products, so structuring systems must be developed to provide vegetable oils with similar characteristics to solid fats. Several ingredients have been used to substitute animal fat by oil emulsions, using ingredients such as konjac, whey protein powder or carrageenan [12,13,14]. In addition, promising results have been reported from the use of oleogels due to its heat resistance and their functionality in carrying lipophilic bioactive substances [15]. Moreover, gel emulsions have shown a great potential to be used as a healthy and low-calorie lipid ingredient [16]. Alginate is widely used in meat product processing due to its properties as a viscosifying, gelling agent and stabilizer [17]. Finally, the use of prebiotic fibers such as inulin with vegetable oils in meat products may improve cookability and reduce hardness compared to the traditional ones [18].

Another novel challenge of the agri-food sector is the integral use of food. About 30% of the food that is produced around the world is lost or wasted along the food supply chain, contributing to environmental pollution and the depletion of natural resources. These include waste in the production, handling, processing or consumption process [19]. Due to the economic, environmental and social significance of food waste, the reduction in the residues generated by food industries is also part of the European Union’s Circular Economy Strategy [20]. In this sense, fourth range industries that produce fruits and vegetables that are ready for consumption, raw or cooked discard large amounts of peels and seeds that still contain valuable compounds that can be reintroduced in the food chain. This is the case of melon and pumpkin seeds, which are considered as residues and discarded in the processing of fourth range products. These seeds contain oil-rich unsaturated fatty acids and other bioactive compounds that can be reintroduced in the food chain to produce healthy foods.

The oil that is extracted from the melon seed has a high nutritional value due to the elevated proportion of essential fatty acids, so it can be used as an alternative source of fat in the food industry [21]. Many health benefits have been reported from the consumption of this oil, since it presents anti-inflammatory and hypoglycaemic properties, and antimicrobial, antigenic and antioxidant potential [22]. The fatty acid pattern of melon seed oil is mainly characterized by the high content in linoleic acid (51–69%), with a proportion of saturated fatty acids lower than 15% [23]. This, together with the presence of other bioactive compounds such as sterols or tocopherols, make it a promising source of fat extracted from industrial waste that can be included in meat products to help to reduce the levels of cholesterol, as well as facilitate a consequent decrease in the development of atherosclerosis related to the incidence of coronary diseases [24]. On the other hand, pumpkin seed oil is also characterized by a high proportion of polyunsaturated fatty acids, ranging from 52.23 to 57.65% [25], where linoleic acid reaches over 44% and is a good source of other phyto-chemicals and bioactive compounds such as tocopherols, β-carotene, squalene and polyphenolic compounds [26]. The presence of such compounds confers pumpkin seed oil a strong antioxidant capacity. In addition, it has shown beneficial effects against benign prostatic hyperplasia [27] and in reducing levels of colorectal, breast, gastric and lung cancer [28].

The aim of this research was to replace the pork fat usually used in the elaboration of deer burgers by several emulsions of melon and pumpkin oils to obtain products with improved nutritional characteristics, especially related to the inclusion of polyunsaturated fatty acids. Texture analyses were performed to evaluate changes caused by the substitution of saturated fats by unsaturated. In addition, nutritional and fatty acids analyses were carried out to verify the improvement in the nutritional characteristics of the burgers made with melon and pumpkin oils. Finally, sensory tests were made to ensure the consumers acceptability of these burgers.

## 2. Materials and Methods

### 2.1. Ingredients and Preparation of Oil Emulsions

Melon (*Cucumis melo* ‘Santa Claus’) and pumpkin (*Cucurbita moschata*) seeds were collected as a residue from an agri-food company dedicated to the production, processing and commercialization of fruits and vegetables (Vicente Peris, S.A., Albuixech, Spain). The seeds were washed to remove the adhered pulp and dried in an oven at 50 ± 2 °C until the moisture showed values below 7%. Then, 5 kg of dried seed from melon and pumpkin, separately, were subjected to oil extraction with a screw press (Komet Oil Press CA59G, IBG Monforts Oekotec GmbH and Co. KG, Mönchengladbach, Germany) at 49 rpm and 100 °C [23]. The oil yield for melon seed oil was 19.44% and for pumpkin seed oil was 22.44%.

To obtain stable emulsions, maltodextrin, inulin, guar gum, xanthan gum and sodium alginate were used (Sosa Ingredients, Barcelona, Spain). Maltodextrin is a gelling agent that gives the oil an emulsion-like texture, although the mixture of maltodextrin and oil is not properly an emulsion since an aqueous phase is not involved. Thus, it was considered like an emulsion because the texture was similar to the other two emulsions elaborated.

Three types of emulsions were carried out (Table 1). The composition of the emulsions was performed according to the use recommendations provided by the manufacturer of the ingredients.

To obtain the Gel and Malto emulsions, the ingredients were mixed at a high speed for 40 s at room temperature, and then stored at 4 °C for 24 h. To obtain the Par emulsion, in addition to homogenizing the mixture of water, oil and alginate at high speed for 20 s, a mixture of water (95%) and calcium chloride (5%) was prepared to help the alginate react and produce the gel. The gel obtained was stored at 4 °C for 24 h.

### 2.2. Elaboration of Burgers

Deer meat was obtained in a local industry specialized in game meat (La Catedral de la Caza S.L., Los Yébenes, Toledo, Spain). In addition, pork fat, thickener (corn-starch) and condiments (garlic, salt and pepper) were purchased from local supermarkets.

**Figure 1:**
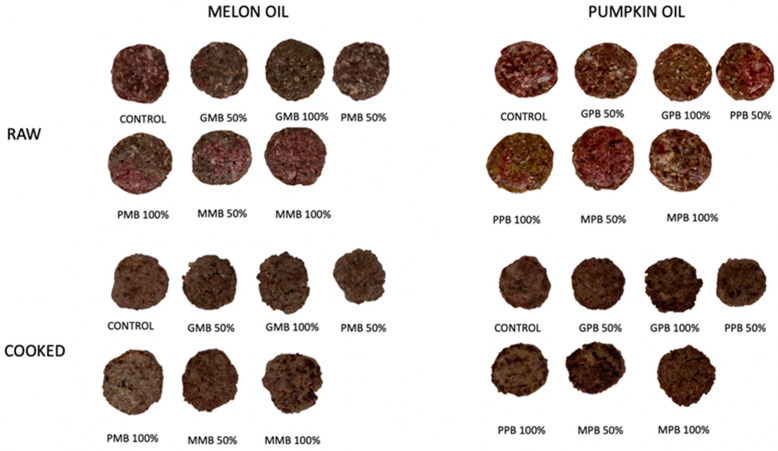
Raw burgers and cooked burgers used for sensory evaluation. Control: Deer meat (78.8%), pork fat (19%), corn-starch (1.4%), spices (0.8%); GMB/GPB 50: Pork fat (9.5%), gel emulsion (9.5%); GMB/GPB 100: Gel emulsion (19%); PMB/PPB 50: Pork fat (9.5%), particle emulsion (9.5%); PMB/PPB 100: Particle emulsion (19%); MMB/MPB 50: Pork fat (9.5%), maltodextrin emulsion (9.5%); MMB/MPB 100: Maltodextrin emulsion (19%). The rest of ingredients in the burgers are the same as the control.

The formulation of the burgers was calculated in order to replace partially (50%) or totally the pork fat used in the traditional recipe of burgers (Table 2). The three oil emulsions (Gel, Par, Malto) were performed for the 2 oils tested (melon and pumpkin), resulting in 12 different batches of burgers (6 of them containing melon oil and 6 containing pumpkin oil). In addition, a control burger was elaborated with pork fat according to the traditional formulation (Table 2, Figure 1). All burgers were made with 78.8% deer meat, 19% fat, 1.4% thickener (maize flour) and 0.8% other condiments (salt, aromatic herbs).

The meat was ground in a grinder (Verder Scientific GmbH & Co. KG, Haan, Germany). The ingredients were mixed, and the burgers were formed with a manual molder of 65 mm diameter with approximately 29 g each. Then, the burgers were stored at −18 °C until the analysis. Before cooking the burgers, they were thawed at 4 °C for 24 h and then roasted in a pan until the central temperature measured with a digital probe thermometer reached 70 °C.

### 2.3. Physical Measurements

Two physical parameters were measured as follows: color and texture. The color was measured in raw burgers with a Minolta CR-200 colorimeter (Minolta Camera Co. Ltd. Osaka, Japan). The measures were taken in five random zones of the surface of four different burgers of each batch using the illuminant D65. The tristimulus values were used to calculate the CIElab* chromatic coordinates: L* (lightness), a* (red-green component), b* (yellow-blue component) [29].

To evaluate the differences in texture, the burgers were subjected to a texture profile analysis test (TPA), in which five samples of each batch were subjected to two consecutive compression tests simulating the chewing process. The average values of the texture parameters hardness, cohesiveness, springiness and chewiness were annotated. The analysis was carried out with a TA-XT Plus texture analyzer (Stable Micro Systems, Godalming, UK). Texture data were obtained from cooked burgers to evaluate differences in the chewing process.

### 2.4. Consumer Preferences

To measure consumers preferences, an affective test with consumer judges was performed. The test was carried out in the sensory analysis laboratory at the Higher Technical School of Agricultural and Forestry Engineering in Albacete (Spain). A nine-point scale ranging from −4 (extremely dislike) to +4 (extremely like) was used. The testing was designed in two different sessions due to the large number of samples to avoid the saturation of the consumer judges [30].

In the first session, the samples corresponding to the burgers formulated with melon seed oil and the control were evaluated by 103 consumers. In the second session, the evaluation was made with the control and the samples that contain pumpkin seed oil. In the second session, the panel was made up of 100 consumers. In both tests, the consumer-judges evaluated the external appearance of raw burgers as well as the smell, flavor and texture of cooked burgers.

### 2.5. Proximate Composition

The proximate composition was performed in cooked burgers. To determine the ash content, samples were calcinated at 550 °C until reaching a constant weight [31]. The protein content was calculated by multiplying the total nitrogen content obtained by the Kjeldahl method by a conversion factor of 6.25 [32]. Crude fat was estimated gravimetrically using the filter bag technique after the petroleum ether extraction of the dried sample in an Ankom XT10 extraction system [33]. For the crude fiber content, the Weende technique adapted to the filter bag technique was used. As described in [33], the Weende technique determines the organic residue remaining after digestion with solutions sodium hydroxide and sulfuric acid by using an Ankom 220 fiber analyzer. Total carbohydrate content was calculated by subtracting the sum of the crude protein, total fat, water and ash from the total weight [34]. Total energy was calculated based on 100 g sample using Atwater values for fat (9 kcal/g), protein (4 kcal/g) and carbohydrate (4 kcal/g) [35].

### 2.6. Fatty Acids

With regards to fatty acid profile, first fat was extracted from burgers with a chloroform-methanol mixture (2:1) according to [36]. Then, fatty acid methyl esters (FAME) were obtained by a transmethylation according to ISO 12988-2:2017 [37]. Then, FAMEs were injected in a Shimadzu GC-2010 Plus Gas Chromatograph (Shimadzu, Tokyo, Japan), equipped with a CPSil 88-fused silica capillary column (50 m × 0.25 mm i.d.), 0.20 m film thickness (Varian, Middelburg, Netherlands), using helium as the carrier gas (120 kPa). Each fatty acid methyl ester (FAME) was identified by direct comparison with a standard mixture (FAME 37, Supelco, Bellefonte, PA, USA). Two samples of each batch were analyzed, and the results were expressed as the percentage of each FAME.

### 2.7. Statistical Analysis

For the analysis of the data, the values obtained from four samples of burgers for each of the physical, nutritional and fatty acid parameters were used. For the analysis of the results obtained in the sensory evaluation, scores of 100 and 103 consumer judges were used. All data are presented as means (n = 4, n = 100, n = 103) and standard deviation (n = 4, n = 100, n = 103). Statistical differences were estimated from an analysis of variance (ANOVA) test at the 5% level of significance and Duncan Test (*p* < 0.05). All statistical analysis were carried out using the SPSS program, release 23.0 for Windows.

## 3. Results

### 3.1. Physical Parameters

Color is one of the most important physical parameters of food since it can determine the preference of consumers for a given product. The inclusion of new ingredients, or the partial or total substitution of these, can cause changes in color that may be strange to the consumer, resulting in product rejection. Therefore, it is important to determine the changes that occur when substituting ingredients in the traditional recipe. In this sense, an objective way to measure color is the use of the CIELab* color space, in which the coordinates L* (lightness), a* (red-green component) and b* (yellow-blue component) are defined.

The inclusion of melon or pumpkin oil in the recipe of burgers resulted in an increase in L* values regardless of the percentage used, with significant differences (*p* < 0.05) respect to control burger (Table 3). Lightness in meat and meat products depends on various factors such as water holding capacity and fat content, as well as the type of ingredients used in the reformulation process [38].

The values obtained for the components a* and b* are shown in Table 3. The control sample presents the highest values of component a* since it does not receive any additional ingredients or has fewer added ingredients that alter the color [39]. Differences between melon and pumpkin burgers may be caused by the different characteristics and composition of the oils [40]. It has been reported that burgers with avocado oil have a greater impact on the color due to the chlorophylls present on the oil, which contribute to the typical green color as it happens in the burgers with pumpkin oil due to the green tones shown [41].

Texture changes are one of the main challenges for the development of meat products with reduced fat and a healthier lipid profile. In meat products made from minced meat, the texture depends on the ability of meat proteins to create gels or the emulsifier capabilities of the non-meat ingredients [42]. Texture parameters (hardness, cohesiveness, springiness, chewiness) can be evaluated by the Texture Profile Analysis, in which two consecutive compressions are applied to the food to simulate chewing process. In general, in all the parameters, the addition of emulsified oils has changed the texture compared to the control sample (Table 4) in a similar way to the results reported by [43] in a study carried out on burgers reformulated with oils and flours from nuts and seeds.

A reduction in the content of animal fat and its substitution by emulsified oils principally affects hardness, resulting in burgers with lower values in this parameter. Similar results have been obtained by [44,45] in beef burgers when fat is substituted by linseed oil or olive oil, also resulting in a decrease in chewiness values. However, it has been reported that the addition of other vegetable oils may lead to an increase in hardness due to the lower fat globule of vegetable oils compared to animal fat, which result in higher protein–protein and protein–lipid interaction [44]. Hardness is supposed to be related to the protein: lipid ratio of the burgers [46], resulting in harder products when the ratio increases. However, in the burgers elaborated with the emulsions of melon and pumpkin oil with guar gum and inulin, sodium alginate, and maltodextrin, hardness was lower independently of the protein: lipid ratio of the burgers. When the percentage of water is increased, it results in a smoother texture, even though the amount of protein is constant [47]. The dilution effect of non-meat ingredients in meat protein systems is primarily responsible for a softer texture [48].

In the rest of the parameters, differences between the control sample with pork fat and the burgers reformulated with vegetable oils were also significant (*p* < 0.05). Due to the high proportion of saturated fatty acids, animal fat that is used in a solid state plays a binding role. Thus, burgers elaborated with vegetables oils usually showed lower values regarding cohesiveness, although when the vegetable oils were textured with calcium alginate (PMB, PPB), the burgers showed similar cohesiveness values to the control. Previous works introducing hydrogelled emulsions of chia or linseed oils elaborated with carrageenan have found no differences in the cohesiveness of burgers compared to the control [46]. Regarding springiness, again the burgers elaborated with vegetable oils showed lower values, probably due to the higher elasticity of the pork fat used in the control burger.

### 3.2. Consumer Preferences

Sensory analysis is a useful tool used to identify the changes originated by the substitution of ingredients based in the preferences of consumers [49]. When the affective test was performed with the burgers elaborated with melon and pumpkin oils, in all cases, and for all the parameters evaluated, the mean values by the consumer judges were above 0, which means that the acceptability of the burgers was in all cases in values of ‘I like’. The samples evaluated are shown in Figure 1.

**Figure 2:**
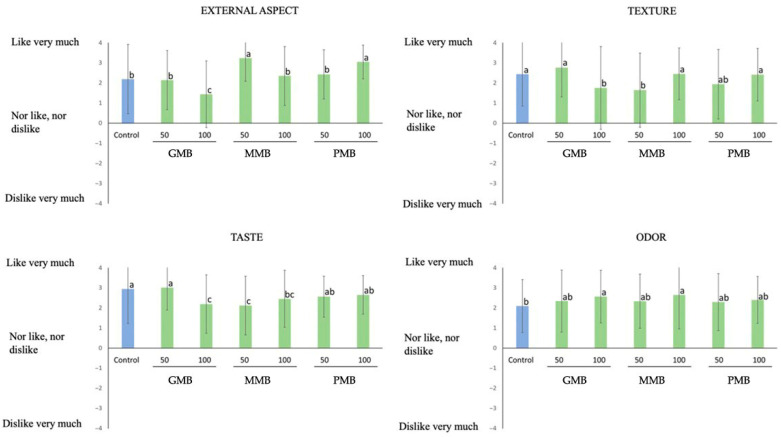
Results obtained in sensory evaluation of reformulated burgers with melon oil. Different letters in the columns show significant differences (*p* < 0.05). Control: Deer meat (78.8%), pork fat (19%), corn-starch (1.4%), spices (0.8%); GMB 50: Pork fat (9.5%), gel emulsion (9.5%); GMB 100: Gel emulsion (19%); PMB 50: Pork fat (9.5%), particle emulsion (9.5%); PMB 100: Particle emulsion (19%); MMB 50: Pork fat (9.5%), maltodextrin emulsion (9.5%); MMB 100: Maltodextrin emulsion (19%). The rest of ingredients in the burgers are the same as the control.

**Figure 3:**
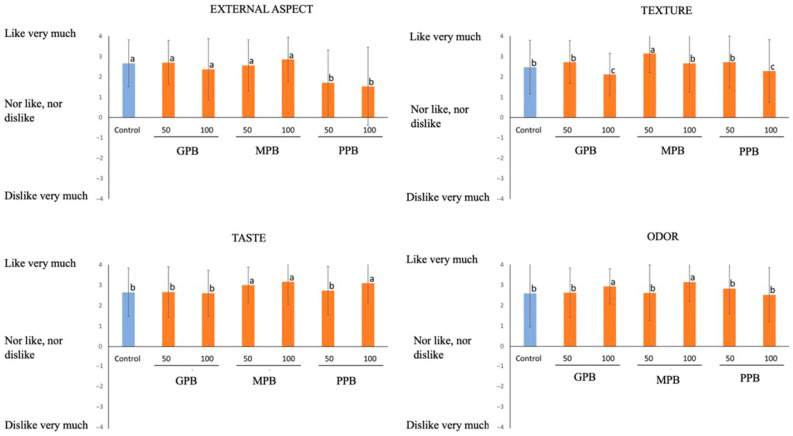
Results obtained in sensory evaluation of reformulated burgers with pumpkin oil. Different letters in the columns show significant differences (*p* < 0.05). Control: Deer meat (78.8%), pork fat (19%), corn-starch (1.4%), spices (0.8%); GPB 50: Pork fat (9.5%), gel emulsion (9.5%); GPB 100: Gel emulsion (19%); PPB 50: Pork fat (9.5%), particle emulsion (9.5%); PPB 100: Particle emulsion (19%); MPB 50: Pork fat (9.5%), maltodextrin emulsion (9.5%); MPB 100: Maltodextrin emulsion (19%). The rest of ingredients in the burgers are the same as the control.

The results of the sensory analysis are shown in Figure 2 for burgers formulated with melon oil and in Figure 3 for the ones formulated with pumpkin oil since they were evaluated in different sessions and by different consumer judges.

No significant differences were found for external aspect when burgers formulated with pumpkin seed oil were evaluated (*p* < 0.05), except PPB burger samples where small green particles could be seen. On the other hand, in the burgers elaborated with melon seed oil, the worst valued sample was GMB 100%, as these burgers seemed the least cohesive of all the samples.

Regarding texture, when this parameter is evaluated by means of sensory evaluation, the burgers with melon and pumpkin oil obtained better or similar results compared to the control sample, specifically in melon seed oil burgers, since GMB 50%, MMB 100%, and PMB 100% did not differ significantly from the control sample (*p* < 0.05). This can be explained because the reformulated burgers have a softer texture [50]. Another factor could be that there is less connective tissue in burgers with less quantity of animal fat [16]. When burgers made up with pumpkin oil were evaluated, MPB 50% obtained the best results due to the juiciness of the sample, in a similar way as reported by [51], where chicken burgers with pumpkin seeds were examined. The worst evaluated on that case was PPB 100% and GPB 100% because they crumbled in the cooking process. The control sample was, in both cases, among the best valued probably because consumers perceived it as the most traditional.

As regards the odor, reformulated burgers with melon seed oil obtained in general better results than the control one. Similar results have been reported by [52] that revealed that fortification of vegetables oils based on ‘emulgels’ improved the odor preferential score of dried fermented deer sausages.

In relation to the pumpkin burgers, the two samples reformulated with pumpkin oil and maltodextrin, as well as the sample with a substitution of 100% of animal fat for particles (PPB 100%), are the ones with the best scores. A study carried out with functional beef burgers formulated with maltodextrin and collagen showed that these were the best at taste acceptance due to the influence of the degree of polymerization of maltodextrin in the retention of volatile flavors [40]. Instead, in the case of melon seed oil, the control sample and the GMB 50% burgers were the best valued, perhaps for being the ones most like the control [53].

### 3.3. Proximate Composition

Table 5 shows the results of the proximate composition of the burgers. The burgers GMB and GPB, formulated with the GEL emulsion (guar gum and inulin), presented the highest protein content (*p* < 0.05). These findings are consistent with those of [54], who stated that there are no differences in raw burgers; however, once cooked, the protein content increases due to the influence of the heat treatments as a result of water loss and the consequent increase in protein content.

Burgers GMB and GPB also showed the highest values in crude fiber, with significant differences from the rest (*p* < 0.05), which may be due to the presence of inulin in the formula, an oligosaccharide with a dietary fiber function [55]. The functional properties of fibers are related to their good effect on human health, as they are associated with the prevention of some diseases such as colon and rectum cancer, abdominal hernias, diabetes, obesity and coronary hearth diseases [56]. As regards the total carbohydrate content, it was found higher in those samples formulated with maltodextrin as it is a polysaccharide.

Related to the fat content, GPB 100 showed the higher values on fats, which was also reported by [57]. In the case of melon seed oil, in GMB 100 the content is lower, and this result can be explained because pumpkin oil presents palmitic oil that is solid at ambient temperature, which can result in a more stable emulsion. During cooking, heat treatment can destabilize emulsions, leading to greater fat loss. In the burgers made with emulsions of melon and pumpkin oils, an excessive loss of fat was not observed during cooking. In this sense, the decrease in fat content can be attributed to the ingredients with which the emulsions were made, which originates a lower amount of fat in the recipe.

### 3.4. Lipid Profile

As regards lipid profile (Table 6 and Table 7), a significant increase in the proportion of linoleic acid (C18:2) was observed in all the samples in which animal fat has been partially or totally replaced by melon or pumpkin oil. In addition, a decrease in the content of palmitic acid (C16:0) was observed. The substitution of animal fat by textured vegetable oils resulted in a decrease in saturated fatty acids and the increase in the concentration of polyunsaturated fatty acids, making products healthier in relation to the incidence of cardiovascular diseases, in which it has been seen that these fatty acids may be involved. Oleic acid (C18:1) is lower in all the modified samples and higher in the control sample. These data agree with the composition of melon seed oil provided by [58], which reported that the main fatty acids found on melon seed oil were linoleic acid (52–69%), oleic acid (12–32%), palmitic acid (9–24%) and stearic acid (5–9%). They also coincide with other studies that indicate that burgers with animal fat contain higher percentages of saturated fatty acids and that those reformulated with vegetable oils have higher percentages of linoleic and oleic acid, depending on the type of oil used in the formulation [59,60].

In pumpkin oil burgers, there was also a decrease in saturated fatty acids (especially palmitic acid) and an increase in the concentration of linoleic acid. In both cases, this effect is generally greater when pumpkin oil is completely substituted for pork fat. This is also due to the lipid composition of pumpkin oil, which is also a rich source of linoleic acid, which has beneficial functions in the human body [61].

## 4. Conclusions

This studys suggest that it is possible to reduce the saturated fat content and increase the polyunsaturated fatty acids proportion in traditional burgers by replacing pork fat with melon and pumpkin seed oil using maltodextrin, alginate and guar gum/inulin emulsions. The total substitution of animal fat by texturized vegetable oils increased the proportion of polyunsaturated fatty acids in burgers, especially the content in linoleic acid, independently of the method used to make the oil emulsions.

The inclusion of vegetable oils produced small changes in the physical characteristics of burgers, since the burgers elaborated with textured vegetable oils showed, in general, a softer and less cohesive texture. The color of raw burgers was also affected, with a reduction in the component a* (red-green component), especially when melon and pumpkin oil was used to totally replace the animal fat contained in the burgers.

From the sensory point of view, all the samples obtained positive evaluations in the affective test performed when flavor, odor, external aspect and texture were evaluated. Even the valuation of some burgers elaborated with melon and pumpkin oils was higher than the control burger elaborated with animal fat in some parameters, showing the good acceptability of these products by consumer judges.

Additionally, the feasibility of using residues from the food industry such as melon and pumpkin seed has been shown considering the stability they have shown in the formation of emulsions, the benefits they have on health based on the fatty acid patterns and by the acceptance of consumers due to the pleasant flavor of both oils.

Future studies may focus on the behavior of the burgers during storage to verify the stability of the burgers and evaluate possible changes originated by the replacement of ingredients.

## Figures and Tables

**Table 1:** Formulation of the three stable emulsions used to replace animal fat.

Ingredient	Emulsion with Guar Gum and Inulin (GEL)	Emulsion with Alginate (PAR)	Emulsion with Maltodextrine (MALTO)
Water	60%	63.5%	0%
Oil	35%	35%	78.5%
Additive	Guar gum (2%), Inulin (3%)	Sodium alginate (0.5%)	Maltodextrine (20.41%), Guar gum (0.78%), Xanthan gum (0.31%)

**Table 2:** Formulation of the burgers elaborated with emulsified melon and pumpkin oil. GMB: Gel emulsion Melon Burger; GPB: Gel emulsion Pumpkin Burger; PMB: Par emulsion Melon Burger; PPB: Par emulsion Pumpkin Burger; MMB: Malto emulsion Melon Burger; MPB: Malto emulsion Pumpkin Burger.

Sample	Deer Meat	Fat	Thickener (Corn Starch)	Condiments
Control	78.8%	Pork fat (19%)	1.4%	0.8%
Burger with Gel emulsion (50%) GMB/GPB	78.8%	Pork fat (9.5%) Gel emulsion (9.5%)	1.4%	0.8%
Burger with Gel emulsion (100%) GMB/GPB	78.8%	Gel emulsion (19%)	1.4%	0.8%
Burger with Par emulsion (50%) PMB/PPB	78.8%	Pork fat (9.5%) Par emulsion (9.5%)	1.4%	0.8%
Burger with Par emulsion (100%) PMB/PPB	78.8%	Par emulsion (19%)	1.4%	0.8%
Burger with Malto emulsion (50%) MMB/MPB	78.8%	Pork fat (9.5%) Malto emulsion (9.5%)	1.4%	0.8%
Burger with Malto emulsion (100%) MMB/MPB	78.8%	Malto emulsion (19%)	1.4%	0.8%

**Table 3:** Color parameters measured in raw samples. L*: Lightness; a*: red-green component; b*: blue-yellow component.

Sample	L*	a*	b*
Control	15.38 ± 0.90 ^a^	15.38 ± 1.5 ^a^	10.39 ± 0.83 ^b^
Melon GMB 50 GMB 100 PMB 50 PMB100 MMB 50 MMB 100	41.74 ± 3.76 ^b^ 45.17 ± 2.71 ^b^ 45.79 ± 4.12 ^b^ 40.70 ± 3.91 ^b^ 48.70 ± 2.92 ^b^ 47.77 ± 3.34 ^b^	14.33 ± 0.72 ^b^ 12.17 ± 0.85 ^c^ 12.49 ± 0.87 ^c^ 11.76 ± 0.82 ^c^ 13.60 ± 0.68 ^b^ 11.39 ± 0.68 ^c^	9.42 ± 0.66 ^b^ 9.22 ± 0.90 ^b^ 9.84 ± 0.69 ^b^ 9.25 ± 0.46 ^b^ 10.19 ± 0.51 ^b^ 9.46 ± 0.85 ^b^
Pumpkin GPB 50 GPB 100 PPB 50 PPB100 MPB 50 MPB 100	40.72 ± 3.92 ^b^ 42.16 ± 3.79 ^b^ 44.77 ± 3.58 ^b^ 42.42 ± 3.39 ^b^ 42.50 ± 2.55 ^b^ 48.11 ± 4.52 ^b^	14.22 ± 1.13 ^b^ 12.59 ± 1.13 ^c^ 12.09 ± 0.85 ^c^ 11.81 ± 0.59 ^c^ 14.20 ± 0.99 ^b^ 10.11 ± 0.61 ^d^	9.42 ± 0.75 ^b^ 10.31 ± 0.93 ^b^ 10.06 ± 0.80 ^b^ 13.10 ± 0.92 ^a^ 10.68 ± 0.64 ^b^ 11.26 ± 0.56 ^b^

Different letters in the columns show significant differences (*p* < 0.05). Control: Deer meat (78.8%), pork fat (19%), corn-starch (1.4%), spices (0.8%); GMB/GPB 50: Pork fat (9.5%), gel emulsion (9.5%); GMB/GPB 100: Gel emulsion (19%); PMB/PPB 50: Pork fat (9.5%), particle emulsion (9.5%); PMB/PPB 100: Particle emulsion (19%); MMB/MPB 50: Pork fat (9.5%), maltodextrin emulsion (9.5%); MMB/MPB 100: Maltodextrin emulsion (19%). The rest of ingredients in the burgers are the same as the control.

**Table 4:** Values for texture parameters in cooked burgers elaborated with melon and pumpkin oils.

Sample	Hardness (g)	Cohesiveness	Springiness	Chewiness (g)
Control	42,858 ± 871 ^a^	0.611 ± 0.67 ^a^	0.771 ± 0.89 ^b^	20,220 ± 525 ^a^
Melon GMB 50 GMB 100 PMB 50 PMB100 MMB 50 MMB 100	32,067 ± 313 ^c^ 15,125 ± 308 ^g^ 36,517 ± 103 ^b^ 35,082 ± 392 ^b^ 30,346 ± 375 ^d^ 26,960 ± 132 ^d^	0.525 ± 0.32 ^b^ 0.445 ± 0.43 ^d^ 0.603 ± 0.60 ^a^ 0.616 ± 0.77 ^a^ 0.552 ± 0.56 ^b^ 0.491 ± 0.31 ^c^	0.690 ± 0.30 ^d^ 0.713 ± 0.76 ^c^ 0.755 ± 0.10 ^b^ 0.785 ± 2.68 ^b^ 0.783 ± 0.91 ^b^ 0.743 ± 1.65 ^c^	11,628 ± 309 ^d^ 4808 ± 100 ^f^ 16,654 ± 625 ^b^ 17,002 ± 811 ^b^ 13,133 ± 194 ^c^ 9849 ± 727 ^d^
Pumpkin GPB 50 GPB 100 PPB 50 PPB100 MPB 50 MPB 100	34,524 ± 459 ^b^ 14,108 ± 511 ^g^ 32,153 ± 488 ^c^ 25,741 ± 334 ^e^ 19,807 ± 639 ^f^ 25,941 ± 423 ^e^	0.550 ± 0.78 ^b^ 0.553 ± 0.57 ^b^ 0.613 ± 0.53 ^a^ 0.637 ± 0.59 ^a^ 0.492 ± 0.56 ^c^ 0.467 ± 0.61 ^d^	0.760 ± 0.57 ^b^ 0.619 ± 1.08 ^f^ 0.701 ± 0.35 ^d^ 0.849 ± 1.01 ^a^ 0.636 ± 0.48 ^e^ 0.617 ± 2.57 ^f^	14,436 ± 206 ^c^ 4665 ± 319 ^f^ 13,808 ± 929 ^c^ 13,947 ± 203 ^c^ 6213 ± 172 ^e^ 7493 ± 675 ^e^

Different letters in the columns show significant differences (*p* < 0.05). Control: Deer meat (78.8%), pork fat (19%), corn-starch (1.4%), spices (0,8%); GMB/GPB 50: Pork fat (9.5%), gel emulsion (9.5%); GMB/GPB 100: Gel emulsion (19%); PMB/PPB 50: Pork fat (9.5%), particle emulsion (9.5%); PMB/PPB 100: Particle emulsion (19%); MMB/MPB 50: Pork fat (9.5%), maltodextrin emulsion (9.5%); MMB/MPB 100: Maltodextrin emulsion (19%). The rest of ingredients in the burgers are the same as the control.

**Table 5:** Proximate composition of the cooked burgers elaborated with melon and pumpkin oil.

Sample	Moisture (%)	Protein (%)	Ash (%)	Crude Fiber (%)	Crude Fat (%)	Total Carbohydrates (%)	Digestive Carbohydrates (%)	Energy Value (Kcal/100 g ms)
Control	1.77 ± 0.12 ^d^	59.31 ± 0.05 ^b^	5.12 ± 0.01 ^b^	0.51 ± 0.01 ^b^	28.27 ± 0.02 ^b^	7.27 ± 0.09 ^d^	6.77 ± 0.01 ^e^	522 ± 1.89 ^b^
Melon GMB 50 GMB 100 PMB 50 PMB100 MMB 50 MMB 100	2.12 ± 0.26 ^b^ 2.67 ± 0.09 ^a^ 2.22 ± 0.18 ^b^ 2.55 ± 0.10 ^a^ 2.05 ± 0.03 ^c^ 2.00 ± 0.14 ^c^	65.42 ± 0.03 ^a^ 63.46 ± 0.02 ^a^ 55.76 ± 0.10 ^c^ 56.79 ± 0.20 ^c^ 56.85 ± 0.04 ^c^ 53.47 ± 0.17 ^d^	5.89 ± 0.06 ^a^ 3.19 ± 0.21 ^c^ 5.32 ± 0.07 ^b^ 5.62 ± 0.11 ^a^ 5.22 ± 0.05 ^b^ 6.00 ± 0.10 ^a^	0.85 ± 0.01 ^a^ 0.95 ± 0.02 ^a^ 0.48 ± 0.02 ^c^ 0.65 ± 0.02 ^b^ 0.53 ± 0.01 ^b^ 0.53 ± 0.01 ^b^	19.32 ± 0.04 ^d^ 29.30 ± 0.07 ^b^ 27.10 ± 0.07 ^b^ 27.07 ± 0.01 ^b^ 23.93 ± 0.01 ^c^ 20.62 ± 0.03 ^d^	9.29 ± 0.04 ^c^ 4.26 ± 0.03 ^e^ 8.14 ± 0.05 ^d^ 10.29 ± 0.04 ^c^ 14.06 ± 0.01 ^b^ 19.51 ± 0.10 ^a^	8.28 ± 0.02 ^d^ 3.66 ± 0.04 ^f^ 7.62 ± 0.02 ^d^ 9.66 ± 0.05 ^c^ 13.45 ± 0.04 ^b^ 18.97 ± 0.03 ^a^	473 ± 3.77 ^e^ 533 ± 1.25 ^b^ 531 ± 0.90 ^b^ 512 ± 1.73 ^c^ 496 ± 3.40 ^d^ 476 ± 2.06 ^e^
Pumpkin GPB 50 GPB 100 PPB 50 PPB100 MPB 50 MPB 100	1.13 ± 0.03 ^f^ 1.51 ± 0.02 ^e^ 1.59 ± 0.03 ^e^ 1.52 ± 0.02 ^e^ 1.83 ± 0.04 ^d^ 1.22 ± 0.10 ^f^	59.31 ± 0.05 ^b^ 61.19 ± 0.51 ^b^ 56.23 ± 0.20 ^c^ 56.56 ± 0.20 ^c^ 52.41 ± 0.20 ^d^ 55.69 ± 0.19 ^c^	5.92 ± 0.05 ^a^ 3.10 ± 0.05 ^c^ 5.77 ± 0.09 ^a^ 5.64 ± 0.11 ^a^ 5.07 ± 0.06 ^b^ 5.41 ± 0.05 ^b^	0.81 ± 0.04 ^a^ 0.93 ± 0.05 ^a^ 0.45 ± 0.05 ^c^ 0.59 ± 0.04 ^b^ 0.61 ± 0.05 ^b^ 0.55 ± 0.04 ^b^	18.57 ± 0.08 ^d^ 32.76 ± 0.22 ^a^ 29.49 ± 0.35 ^b^ 27.60 ± 0.26 ^b^ 18.58 ± 0.12 ^d^ 26.94 ± 0.02 ^b^	7.27 ± 0.09 ^d^ 7.94 ± 0.26 ^d^ 8.08 ± 0.12 ^d^ 9.97 ± 0.31 ^c^ 15.04 ± 0.14 ^b^ 20.22 ± 0.14 ^a^	6.77 ± 0.01 ^e^ 8.57 ± 0.13 ^d^ 7.61 ± 0.22 ^d^ 9.41 ± 0.17 ^c^ 14.86 ± 0.14 ^b^ 19.58 ± 0.27 ^a^	522 ± 1.89 ^b^ 563 ± 6.55 ^a^ 525 ± 6.02 ^b^ 513 ± 4.08 ^c^ 507 ± 2.87 ^c^ 470 ± 3.68 ^e^

Different letters in the columns show significant differences (*p* < 0.05). Control: Deer meat (78.8%), pork fat (19%), corn-starch (1.4%), spices (0.8%); GMB/GPB 50: Pork fat (9.5%), gel emulsion (9.5%); GMB/GPB 100: Gel emulsion (19%); PMB/PPB 50: Pork fat (9.5%), particle emulsion (9.5%); PMB/PPB 100: Particle emulsion (19%); MMB/MPB 50: Pork fat (9.5%), maltodextrin emulsion (9.5%); MMB/MPB 100: Maltodextrin emulsion (19%). The rest of ingredients in the burgers are the same as the control.

**Table 6:** Fatty acid composition of the burgers reformulated with melon seed oil.

Fatty Acid	Control	GMB 50	GMB 100	PMB 50	PMB 100	MMB 50	MMB 100
C12:0 C14:0 C16:0 C16:1 C17:0 C18:0 C18:1 C18:2 C18:3 C20:0 C20:1	0.13 ± 0.05 2.01 ± 0.07 23.87 ± 0.17 ^a^ 3.17 ± 0.03 0.55 ± 0.06 13.81 ± 0.09 ^a^ 41.56 ± 0.17 ^a^ 12.56 ± 0.12 ^e^ 0.76 ± 0.11 0.04 ± 0.01 0.93 ± 0.05	0.08 ± 0.01 1.56 ± 0.05 16.61 ± 0.12 ^c^ 2.15 ± 0.06 0.38 ± 0.55 12.71 ± 0.24 ^b^ 33.57 ± 0.18 ^b^ 26.73 ± 0.36 ^c^ 0.70 ± 0.05 0.08 ± 0.01 0.52 ± 0.05	0.08 ± 0.01 2.20 ± 0.10 21.17 ± 0.11 ^b^ 2.47 ± 0.09 0.21 ± 0.03 12.07 ± 0.24 ^b^ 28.38 ± 0.09 ^d^ 32.29 ± 0.12 ^b^ 0.54 ± 0.06 0.14 ± 0.03 0.19 ± 0.02	0.06 ± 0.01 1.34 ± 0.06 20.42 ± 0.48 ^b^ 1.67 ± 0.02 2.14 ± 0.06 14.59 ± 0.05 ^a^ 33.6 ± 0.12 ^b^ 28.40 ± 0.09 ^c^ 0.49 ± 0.10 0.01 ± 0.00 0.44 ± 0.06	0.03 ± 0.01 0.75 ± 0.11 18.21 ± 0.44 ^c^ 1.55 ± 0.05 0.13 ± 0.01 12.00 ± 0.08 ^b^ 31.62 ± 0.16 ^b^ 37.45 ± 0.09 ^a^ 0.35 ± 0.04 0.02 ± 0.00 0.36 ± 0.03	0.08 ± 0.01 1.84 ± 0.05 17.63 ± 0.08 ^c^ 2.20 ± 0.09 1.64 ± 0.08 8.35 ± 0.09 ^c^ 30.65 ± 1.43 ^c^ 22.35 ± 0.17 ^d^ 0.63 ± 0.08 0.04 ± 0.00 0.33 ± 0.05	0.05 ± 0.01 1.09 ± 0.02 13.51 ± 0.30 ^d^ 1.55 ± 0.05 0.18 ± 0.02 4.16 ± 0.07 ^d^ 29.79 ± 0.06 ^c^ 27.25 ± 0.10 ^c^ 0.57 ± 0.05 0.03 ± 0.00 0.15 ± 0.03
SFA MUFA PUFA	40.41 ± 0.12 45.66 ± 0.18 13.32 ± 0.04	31.42 ± 0.15 36.24 ± 0.07 27.43 ± 0.05	35.87 ± 0.18 31.04 ± 0.06 32.83 ± 0.13	38.56 ± 0.11 35.71 ± 0.18 28.89 ± 0.06	31.14 ± 0.15 33.53± 0.007 37.80 ± 0.18	29.58 ± 0.12 33.18 ± 0.09 22.98 ± 0.11	19.02 ± 0.04 31.49 ± 0.06 27.82 ± 0.14

SFA: Saturated fatty acids; MUFA: Monounsaturated fatty acids; PUFA: Polyunsaturated fatty acids. Different letters in the rows show significant differences (*p* < 0.05).Control: Deer meat (78.8%), pork fat (19%), corn-starch (1.4%), spices (0,8%); GMB 50: Pork fat (9.5%), gel emulsion (9.5%); GMB 100: Gel emulsion (19%); PMB 50: Pork fat (9.5%), particle emulsion (9.5%); PMB 100: Particle emulsion (19%); MMB 50: Pork fat (9.5%), maltodextrin emulsion (9.5%); MMB 100: Maltodextrin emulsion (19%). The rest of ingredients in the burgers are the same as the control.

**Table 7:** Fatty acid composition of the burgers reformulated with pumpkin seed oil.

Fatty Acid	Control	GPB 50	GPB 100	PPB 50	PPB 100	MPB 50	MPB 100
C12:0 C14:0 C16:0 C16:1 C17:0 C18:0 C18:1 C18:2 C18:3 C20:0 C20:1	0.13 ± 0.05 2.01 ± 0.07 23.87 ± 0.17 ^a^ 3.17 ± 0.03 0.55 ± 0.06 13.81 ± 0.09 ^a^ 41.56 ± 0.17 ^a^ 12.56 ± 0.12 ^f^ 0.76 ± 0.11 0.04 ± 0.01 0.93 ± 0.05	0.08 ± 0.01 1.81 ± 0.11 18.67 ± 0.08 ^b^ 2.32 ± 0.14 0.43 ± 0.04 11.80 ± 0.24 ^b^ 31.05 ± 0.19 ^c^ 38.00 ± 0.14 ^b^ 0.45 ± 0.17 0.14 ± 0.04 0.25 ± 0.04	0.09 ± 0.01 2.04 ± 0.04 16.39 ± 0.08 ^c^ 2.07 ± 0.01 0.35 ± 0.06 13.45 ± 0.15 ^a^ 33.51 ± 0.10 ^b^ 27.40 ± 0.12 ^d^ 0.51 ± 0.06 0.01 ± 0.00 0.52 ± 0.06	0.10 ± 0.02 2.11 ± 0.03 19.24 ± 0.08 ^b^ 2.41 ± 0.18 0.26 ± 0.01 13.81 ± 0.02 ^a^ 33.69 ± 0.24 ^b^ 27.70 ± 0.14 ^d^ 0.60 ± 0.01 0.04 ± 0.00 0.44 ± 0.02	0.07 ± 0.00 1.62 ± 0.20 15.03 ± 0.11 ^c^ 1.35 ± 0.01 0.26 ± 0.03 12.67 ± 0.09 ^b^ 31.45 ± 0.15 ^c^ 34.25 ± 0.17 ^c^ 0.45 ± 0.04 0.03 ± 0.00 0.23 ± 0.01	0.12 ± 0.01 2.11 ± 0.03 19.24 ± 0.04 ^b^ 2.46 ± 0.02 0.26 ± 0.01 10.54 ± 0.08 ^c^ 40.74 ± 0.09 ^a^ 23.91 ± 0.03 ^e^ 0.55 ± 0.02 0.04 ± 0.00 0.55 ± 0.01	0.04 ± 0.00 0.84 ± 0.04 12.70 ± 0.08 ^d^ 0.74 ± 0.03 0.18 ± 0.01 9.83 ± 0.05 ^c^ 31.80 ± 0.12 ^c^ 43.58 ± 0.03 ^a^ 0.36 ± 0.03 0.04 ± 0.01 0.19 ± 0.01
SFA MUFA PUFA	40.41 ± 0.12 45.66 ± 0.18 13.32± 0.04	32.93 ± 0.08 33.62 ± 0.10 38.45± 0.13	32.33 ± 0.13 36.10 ± 0.07 27.91 ± 0.11	35.56 ± 0.14 36.54 ± 0.14 28.3 ± 0.12	29.68 ± 0.13 33.03 ± 0.14 34.70 ± 0.12	32.31 ± 0.11 43.75 ± 0.09 24.46 ± 0.06	23.63 ± 0.09 32.73 ± 0.08 43.94 ± 0.09

SFA: Saturated fatty acids; MUFA: Monounsaturated fatty acids; PUFA: Polyunsaturated fatty acids. Different letters in the rows show significant differences (*p* < 0.05). Control: Deer meat (78.8%), pork fat (19%), corn-starch (1.4%), spices (0.8%); GPB 50: Pork fat (9.5%), gel emulsion (9.5%); GPB 100: Gel emulsion (19%); PPB 50: Pork fat (9.5%), particle emulsion (9.5%); PPB 100: Particle emulsion (19%); MPB 50: Pork fat (9.5%), maltodextrin emulsion (9.5%); MPB 100: Maltodextrin emulsion (19%). The rest of ingredients in the burgers are the same as the control.

## Data Availability

The data presented in this study are available on request from the corresponding author.
